# Dissecting the Crosstalk Between Nrf2 and NF-κB Response Pathways in Drug-Induced Toxicity

**DOI:** 10.3389/fcell.2021.809952

**Published:** 2022-02-02

**Authors:** Wen Gao, Lin Guo, Yan Yang, Yu Wang, Shuang Xia, Hui Gong, Bi-Kui Zhang, Miao Yan

**Affiliations:** ^1^ Department of Pharmacy, The Second Xiangya Hospital, Central South University, Changsha, China; ^2^ Xiangya School of Medicine, Central South University, Changsha, China

**Keywords:** Nrf2, NF-κB, crosstalk, drug-induced toxicity, toxic reactions

## Abstract

Nrf2 and NF-κB are important regulators of the response to oxidative stress and inflammation in the body. Previous pharmacological and genetic studies have confirmed crosstalk between the two. The deficiency of Nrf2 elevates the expression of NF-κB, leading to increased production of inflammatory factors, while NF-κB can affect the expression of downstream target genes by regulating the transcription and activity of Nrf2. At the same time, many therapeutic drug-induced organ toxicities, including hepatotoxicity, nephrotoxicity, cardiotoxicity, pulmonary toxicity, dermal toxicity, and neurotoxicity, have received increasing attention from researchers in clinical practice. Drug-induced organ injury can destroy body function, reduce the patients’ quality of life, and even threaten the lives of patients. Therefore, it is urgent to find protective drugs to ameliorate drug-induced injury. There is substantial evidence that protective medications can alleviate drug-induced organ toxicity by modulating both Nrf2 and NF-κB signaling pathways. Thus, it has become increasingly important to explore the crosstalk mechanism between Nrf2 and NF-κB in drug-induced toxicity. In this review, we summarize the potential molecular mechanisms of Nrf2 and NF-κB pathways and the important effects on adverse effects including toxic reactions and look forward to finding protective drugs that can target the crosstalk between the two.

## 1 The Overview of Nuclear Factor Erythroid (NF-E2)-Related Factor 2 (Nrf2) and Nuclear Factor-Kappa B (NF-κB)

### 1.1 The Role of Nrf2 and Its Regulation

When our body is continuously exposed to external stresses, it’s easy to produce the excessive free radicals and reactive oxygen species (ROS) in the mitochondria, peroxisomes, and endoplasmic reticulum (ER), and downregulate endogenous antioxidants such as enzymes and antioxidant proteins (may be stress upregulated sometimes, but not enough to resist oxidative stress), thereby leading to damage of cellular components including proteins, DNA, and lipids (Tonelli et al., 2018). Antioxidant proteins are substances that can protect cells against free radical damage. However, supplementation of exogenous antioxidants can target oxidative stress by inhibiting the production of free radicals and ROS and bolstering the endogenous antioxidant capacity. Originally characterized as a master regulator of oxidative stress, the transcription factor Nrf2 continues to emerge as a critical mediator of cellular defense. Through the ongoing study on the Nrf2 pathway, more researchers find that Nrf2 is beneficial for cell survival and proliferation from redox homeostasis, drug/xenobiotic metabolism to DNA repair (Dodson et al., 2019).

Nrf2 is a key transcription factor with a basic-region leucine zipper (bZIP) structure in the Cap-n-Collar (CNC) family, which controls many aspects of cell homeostasis in response to oxidative stress and toxic insults in various tissues and cells (Buendia et al., 2016; Skibinski et al., 2017; Dinkova-Kostova et al., 2018; Qu et al., 2020). It is a modular protein composed of seven Nrf2-ECH homology domains (Neh1-7) that perform different functions (Hayes and Dinkova-Kostova, 2014). Neh2 contains two important motifs known as DLG and ETGE, which are essential for the interaction between Nrf2 and its negative regulator Kelch-like ECH-associated protein 1 (Keap1) (Wang et al., 2014; Canning et al., 2015). The synthesized Nrf2 will translocate to the nucleus, and then form heterodimers with small musculoaponeurotic fibrosarcoma protein (MAF) K, G, and F. The heterodimer recognizes an enhancer sequence termed antioxidant response element (ARE) which is located in the regulatory regions of cellular defense enzyme genes. The target genes of Nrf2 encode the antioxidant proteins, metabolic enzymes, transporters, redox balancing factors, and glutathione-conjugated coenzyme (Ma, 2013; Hayes and Dinkova-Kostova, 2014; Wang et al., 2014; Canning et al., 2015; Cuadrado et al., 2018).

### 1.2 Keap1-Dependent Nrf2 Regulation

**FIGURE 1:**
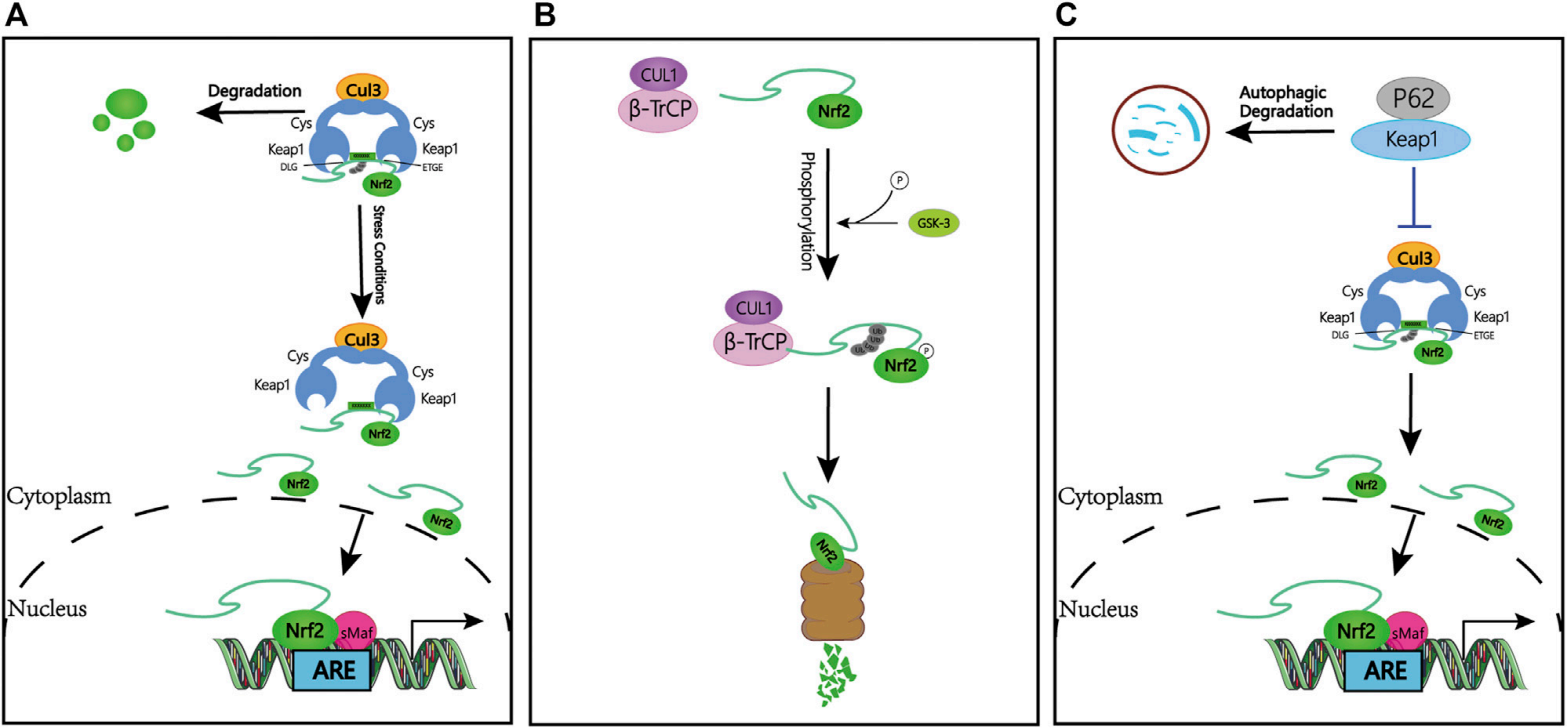
Keap1-dependent and -independent regulation of Nrf2. **(A)** Keap1-dependent Nrf2 regulation. Under normal conditions, Nrf2 is sequestered in the cytoplasm by Keap1. Under oxidative stress, Nrf2 dissociates from Keap1, translocates to the nucleus and activates the ARE-gene battery. **(B)** Keap1-independent Nrf2 regulation. Phosphorylation of Nrf2 by GSK facilitates the recognition of Nrf2 by β-TrCP for CUL1-mediated ubiquitination and subsequent proteasome degradation. **(C)** P62-dependent Nrf2 regulation. p62 is sequestered with Keap1 and increased Nrf2 signaling. Abbreviations: Keap1, Kelch-like ECH-associated protein 1; Nrf2, Nuclear Factor Erythroid (NF-E2)-Related Factor 2; ARE, antioxidant response element; GSK-3, Glycogen synthase kinase 3; β-TrCP, β-transducin repeat-containing protein; Ub, ubiquitin.

In the review of previous studies, the regulation of Nrf2 is considered a beneficial target for patients to release the disease. The best-characterized mechanism of Nrf2 regulation is the control of protein stabilization by the keap1-Cullin3 (Cul3)- Ring-box protein 1 (Rbx1) complex, which acts as a protein level rheostat. Keap1 binds to Nrf2 *via* a specific amino acid sequence on its homodimeric N-terminal domain, including siting with low (aspartate, leucine, and glycine; DLG) and high (glutamate, threonine, glycine, and glutamate; ETGE) affinity (hinge and latch hypothesis) (Cuadrado et al., 2018). Under the physiological state, Keap1 binds to Nrf2 and promotes ubiquitination and subsequent proteasomal degradation (Suzuki et al., 2013; Tonelli et al., 2018). Due to the short half-life of Nrf2 (10–30 min), Nrf2 remains at a very low basal level by sustained inhibition of Keap1. When stimulated by oxidative stress or Nrf2 activators, the thiol groups on cysteine residues in Keap1 are modified by electrophiles, and the function of Keap1 is diminished, ultimately resulting in the dissociation of Nrf2 from Keap1 and activation of Nrf2 and ARE genes (Hayes et al., 2010). Studies show that products of the Nrf2/ARE pathway, including NADPH, NAD (P) H quinone oxidoreductase 1 (NQO1), glutathione peroxidase, and heme oxygenase 1 (HO-1), protect cells from various injuries and enhance body resistance through their activation (Ahmed et al., 2017) (Figure 1A).

### 1.3 Keap1-independent Nrf2 Regulation

Interestingly, Keap1-independent Nrf2 regulation is achieved through Glycogen synthase kinase 3 (GSK-3)/β-transducin repeat-containing protein (β-TrCP). β-TrCP is a substrate receptor for the S-phase kinase-associated protein 1 (Skp1)-Cul1-Rbx1/Roc1 ubiquitin ligase complex that targets Nrf2 for ubiquitination and proteasomal degradation (Chowdhry et al., 2013). GSK-3 is responsible for phosphorylating a domain of Nrf2 (aspartate, serine, glycine, isoleucine, serine; DSGIS), creating a recognition motif for β-TrCP and then presenting Nrf2 to the Skp1-Cul1-Rbx1/Roc1 complex, finally leading to an alternative pathway for inhibition of Nrf2 (Rada et al., 2011; Cuadrado, 2015) (Figure 1B).

### 1.4 P62-dependent Nrf2 κRegulation

Additional pieces of evidence suggest that other regulators also may activate the Nrf2 pathway. P62, for example, sequesters Keap1 to its autophagic degradation and ultimately activates Nrf2-dependent genes (Ma, 2013; Ahmed et al., 2017) (Figure 1C).

## 2 The NF-κB Cellular Function and regulation

The NF-κB family, a regulator of κB light chain expression in mature B and plasma cells, regulates many genes involved in different cellular processes, such as cell differentiation, proliferation, development, and apoptosis. This family is composed of five structurally related members NF-κB1 (also called p50), NF-κB2 (also called p52), RelA (also called p65), c-Rel, and RelB, encoded by NF-κB1, NF-κB2, RELA, REL, and RELB, respectively. They share an N-terminal Rel homology domain (RHD) which is responsible for sequence-specific DNA binding, and homo- and hete-rodimerization (Hayden and Ghosh, 2008; Liu et al., 2017a; Peng et al., 2020). Based on the different mediators in the signaling cascade, the NF-κB signaling pathway can be categorized as either a canonical or noncanonical (alternative) pathway.

### 2.1 Canonical Pathway

**FIGURE 2:**
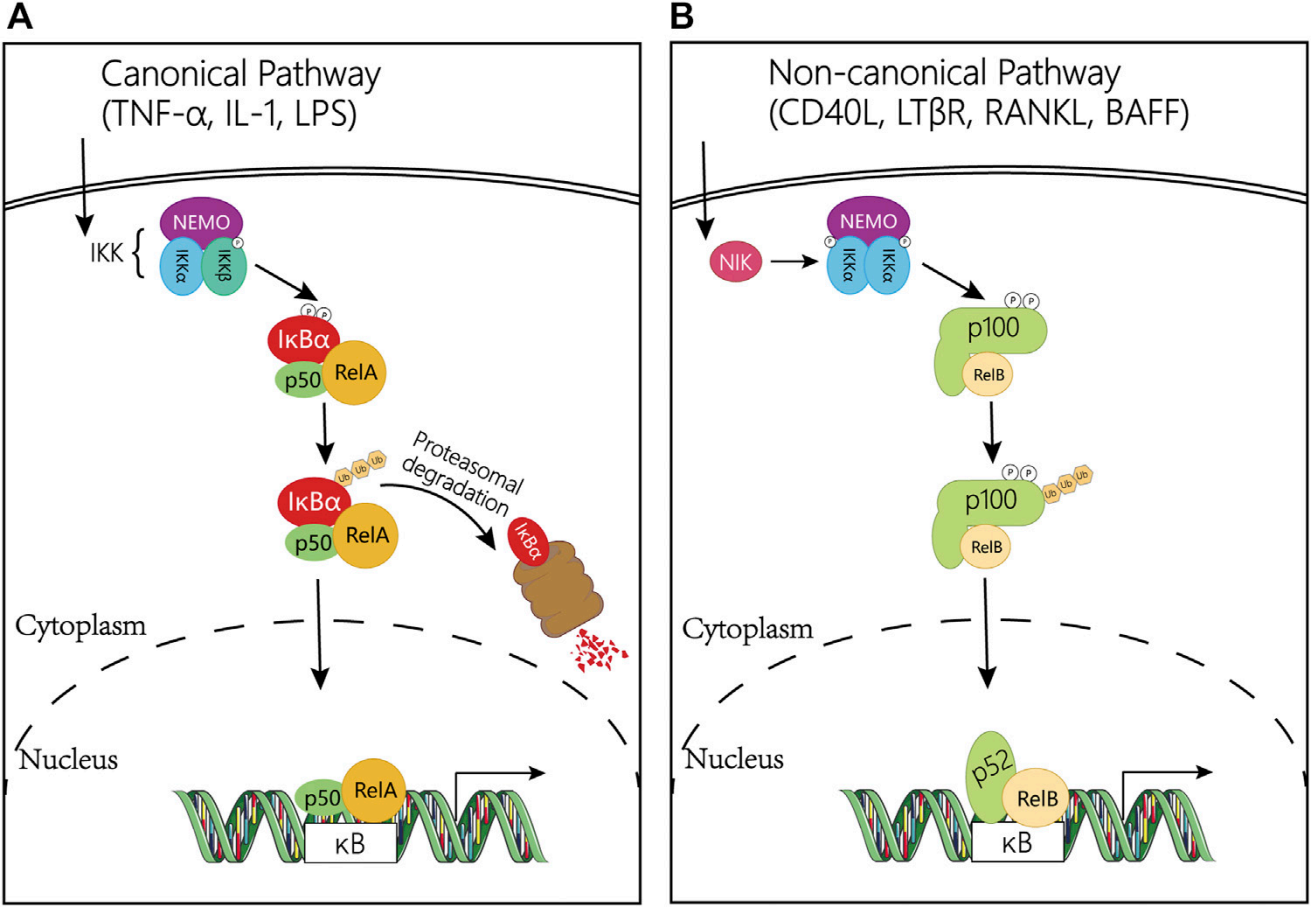
Regulation mechanism diagram of NF-κB pathway. (**A)** Canonical NF-κB signaling pathway. The classical pathway is triggered by TNFα, IL-1, or LPS, and subsequently activates the IκK complex, which induces phosphorylation of IκBα and promotes its degradation. This leads to the release of the NF-κB heterodimer RelA/p50, which then translocates to the nucleus and induces the transcription of target genes. (**B)** Non-canonical NF-κB signaling pathway. This pathway is activated by CD40L, LTβR, RANKL and BAFF gene, and mediated by NIK and the IκK complex containing two IκKα subunits, but not NEMO. In the non-canonical pathway, receptor binding leads to activation of the NF-κB-inducible kinase NIK, which phosphorylates and activates the IκKα complex, which in turn phosphorylates two serine residues adjacent to the C-terminal IκB domain of the p100 ankyrin repeat, resulting in its partial proteolysis and release of the p52/RelB complex. Abbreviations: NF-κB, Nuclear Factor-kappa B; TNFα: tumor necrosis factor α; IL-1: interleukin-1; LPS: lipopolysaccharide; IκB, an inhibitor of κB; IκK, IκB kinase; CD40L, CD40 ligand; RANKL, receptor activator of nuclear factor kappa-B ligand; LTβR, lymphotoxin beta; BAFF, B cell-activating factor; NIK, NF-κB inducing kinase.

Activation of the canonical NF-κB pathway relies on its ability to bind with DNA controlled by the inhibitor of κB (IκB). IκB protein, a subfamily of the large ankyrin repeat domain (ARD) containing superfamily, can bind NF-κB dimers to prevent the activation of NF-κB. The most common member of IκB in mediating the activation of the typical NF-κB pathway is IκBα, which plays a central role in subsequent transcription (Hayden and Ghosh, 2008). Degradation of IκBα involves phosphorylation of IκB kinase (IκK) which is a trimeric complex composed of two catalytic subunits, IκKα and IκKβ, and a regulatory subunit, IκKγ (also named NF-κB essential modulator or NEMO) (Sun and Ley, 2008). In response to external stimulation, NF-κB-bound IκB protein undergoes specific phosphorylation, ubiquitination, and proteasome-mediated proteolysis, allowing NF-κB to localize to the nucleus and bind to DNA (Hoffmann et al., 2006; Siggers et al., 2011; Wang et al., 2012; Zhao et al., 2014; Cildir et al., 2016) (Figure 2A).

### 2.2 Non-Canonical Pathway

On the contrary, activation of the non-canonical NF-κB pathway is slow and depend on a different set of protein synthesis, including CD40 ligand (CD40L), receptor activator of nuclear factor kappa-B ligand (RANKL), lymphotoxin beta (LTβR), and B cell-activating factor (BAFF) (Sun and Liu, 2011; Sun, 2012). In addition, the non-canonical NF-κB pathway relies on the processing of the NF-κB2 precursor protein, p100 rather than the degradation of IκBα. The NF-κB inducing kinase (NIK) phosphorylates and activates IκKα, then synergistically regulating the phosphorylation of p100 (Senftleben et al., 2001; Sun, 2011; Sun, 2012). After that, this phosphorylation in turn induces the ubiquitination and proteasome-mediated partial degradation of p100 to generate p52. Meanwhile, the degradation processing of p100 not only forms p52 but also allows the nuclear translocation of the noncanonical NF-κB complex p52/RelB and then activates the NF-κB (Senftleben et al., 2001; Xiao et al., 2001; Sun, 2012; Cildir et al., 2016; Liu et al., 2017a). In the nucleus, p65 coordinates gene transcription by recruiting coactivators (e.g., CREB-binding proteins (CBP)) or corepressors (e.g., histone deacetylases (HDACs)) (Liu et al., 2008) (Figure 2B).

In the review of previous clinical and experimental studies, we find that tissues after external stimulation secrete cytokines including interleukin-1 (IL-1) and tumor necrosis factor-alpha (TNF-α), of which TNF-α stimulates the activation of other inflammatory mediators (including NF-κB), resulting in the release of inflammatory genes, and then leading to the persistence and deterioration of inflammation (Liu, 2005; Ju et al., 2020).

## 3 The Interplay Between Nrf2 and NF-κB Pathways

The Nrf2 and NF-κB pathways co-regulate cellular responses to oxidative stress and inflammation. The underlying complex molecular mechanisms between these two important pathways remain to be further elucidated.

### 3.1 NF-κB Inhibits the Nrf2 Pathway

**FIGURE 3:**
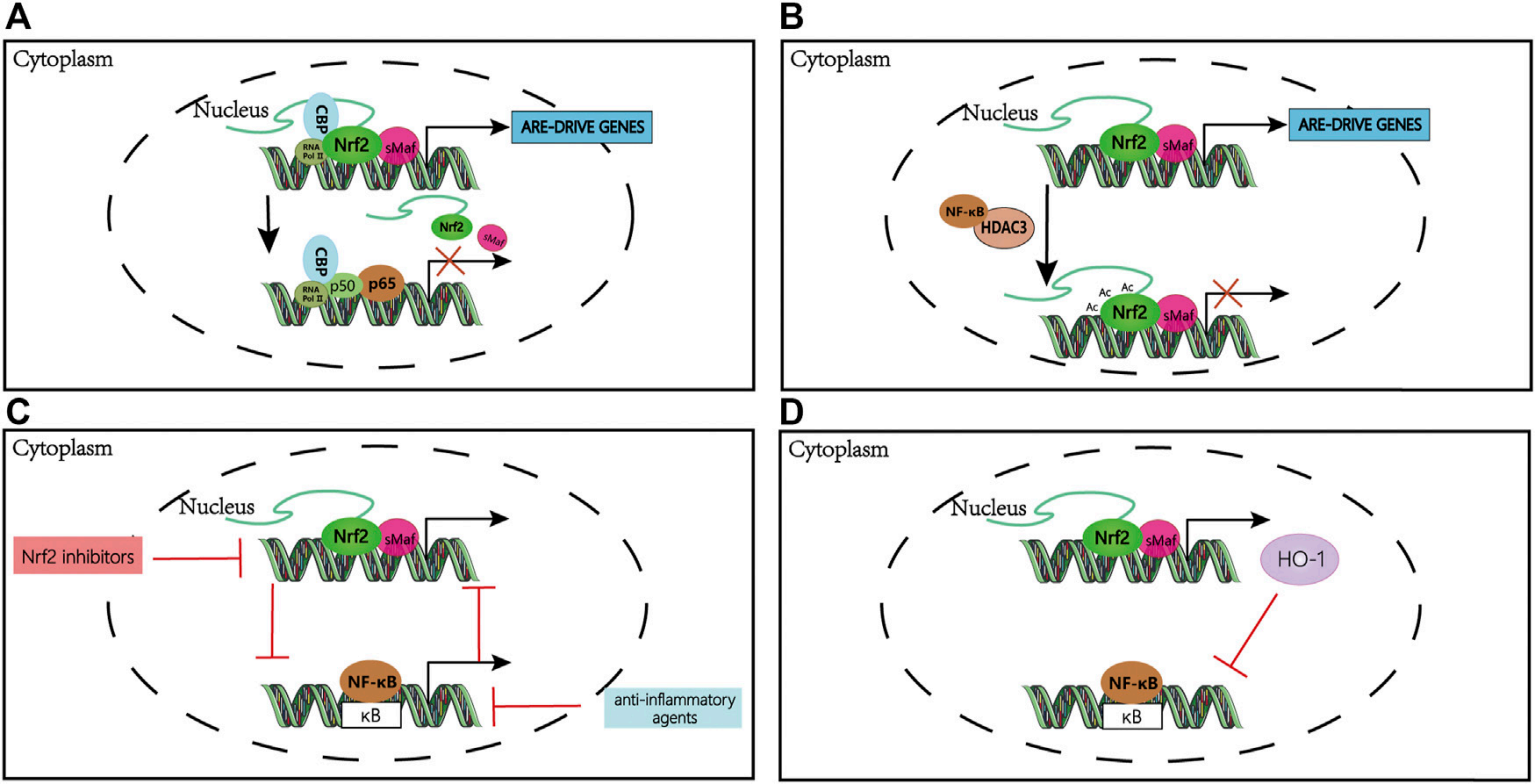
The mechanism of crosstalk between Nrf2 and NF-κB pathways. **(A)** Nrf2 and NF-κB compete for CBP binding in the nucleus; binding of either transcription factor to CBP is dependent on the relative amount of translocated Nrf2 and NF-κB. (**B)** NF-κB-recruited HDAC3 deacetylates Nrf2 inhibiting ARE-dependent gene expression. (**C)** Nrf2 is indirectly activated by anti-inflammatory compounds that suppress NF-κB activity; likewise, NF-κB is indirectly activated by Nrf2 inhibitors. (**D)** HO-1, a downstream gene of Nrf2, can inhibit NF-κB transcription. Abbreviations: CBP, CREB-binding proteins; HDACs, histone deacetylases; Ac, deacetylation; HO-1, heme oxygenase 1.

The NF-κB p65 subunit can directly inhibit the Nrf2 pathway at the transcriptional level. On the one hand, the NF-κB p65 subunit competes with Nrf2 for the CH1-KIX domain of the transcriptional co-activator CBP, leading to the inactivation of the Nrf2 pathway (Figure 3A). On the other hand, NF-κB recruits HDAC3 by promoting the interaction of HDAC3 with CBP or MafK, causing local hypoacetylation and impeding Nrf2 signaling (Liu et al., 2008; Bellezza et al., 2010) (Figure 3B). Interestingly, a large number of anti-inflammatory agents can activate the Nrf2 pathway by interfering with NF-κB activation (Li et al., 2008; Minelli et al., 2012; Kim et al., 2013; Grottelli et al., 2016).

### 3.2 Nrf2 Inhibits the NF-κB Pathway

Nrf2 deficient mice subjected to severe head injury showed higher NF-κB activity compared to wild-type Nrf2 mice (Jin et al., 2008) (Figure 3C). A study explored the effect of Nrf2 on the activation of NF-κB and expression of inflammatory cytokine by using primary cultured astrocytes from Nrf2 wild-type (WT) or knockout (KO). The activity of NF-κB and the expression of inflammatory cytokines including TNF-α, IL-1β, interleukin-6 (IL-6), and matrix metallopeptidase9 (MMP9) were upregulated in Nrf2 KO astrocytes and WT astrocytes, but the increase of which was more apparent in Nrf2 KO astrocytes. These results suggest that loss of Nrf2 may induce more aggressive inflammation by activating NF-κB and downstream pro-inflammatory cytokines (Pan et al., 2012). However, NF-κB-DNA-binding activity was significantly inhibited in a diabetic mouse model with Nrf2 overexpression (Song et al., 2009). These studies suggest that we should also notice the negative regulation of NF-κB by Nrf2.

There are results indicated that the Nrf2 gene has an NF-κB binding site which p65/p50 heterodimer is recruited, and IκK-β plays a core role in NF-κB signaling, contains an ETGE motif that enables it to bind to Keap1 (Lee et al., 2009; Jiang et al., 2013)**.** Thereby, the selective recognition mechanism of Keap1 with IκKβ or Nrf2 is vital to the crosstalk between NF-κB and Nrf2 signaling. Besides, the Rho GTPase protein (RAC1) through IκBα induces the increase of the transcriptional level of NF-κB, which in turn up-regulates the expression of Nrf2. However, Nrf2 deficiency induces IκBα phosphorylation and subsequent degradation, increasing NF-κB levels and inducing inflammation (Cuadrado et al., 2014). Collectively, Nrf2 inhibits NF-κB as a regulatory feedback loop. Moreover, much evidence indicates that Nrf2 downstream gene HO-1, one of the centers of the crosstalk between Nrf2 and NF-κB, plays a vital role in regulating inflammatory responses (Figure 3D). HO-1 may inhibit the nuclear translocation of the NF-κB pathway to mediate inflammation *via* its end-products, i.e., carbon monoxide and bilirubin which have been reported to prevent the activation of NF-κB (Lee et al., 2003; Rushworth et al., 2008; Bellezza et al., 2012). Also, a study demonstrates that HO-1 inhibits LPS-induced TNF and IL-1β expression through suppression of NF-κB activation (Rushworth et al., 2008).

## 4 Toxic Reactions

### 4.1 Hepatotoxicity

The liver is an important central organ for metabolism and detoxification in the human body and is susceptible to many factors, such as excessive alcohol consumption, hepatoviruses, various toxins, and drugs, leading to the development of liver diseases. Among them, drug-induced liver injury (DILI) and liver failure have high morbidity and mortality worldwide and it deserves further to explore the mechanisms and new targets against drug-induced hepatotoxicity (Andrade et al., 2019). Transcriptomic analysis of a variety of DILI compounds in primary hepatocytes showed that NF-κB inhibition coincided with a strong Nrf2 stress response, which might sensitize hepatocytes to pro-apoptotic signaling cascades induced by endogenous cytotoxic proinflammatory cytokines and play a key role in the regulation of DILI (Herpers et al., 2016).

Nrf2 signaling pathway is an important way to regulate cellular oxidative stress, which plays an important role in DILI (Tong et al., 2006). Various regulatory factors are affecting the Nrf2 pathway. A study suggests that intestinal microorganisms modulate hepatic susceptibility to oxidative injury *via* the Nrf2 signaling pathway (Saeedi et al., 2020). Also, anti-inflammatory agents contribute a lot against hepatotoxicity.

#### 4.1.1 Acetaminophen (APAP)-Induced Hepatotoxicity

Most DILI is caused by an intentional or unintentional overdose of APAP, which accounts for approximately 50% of all cases of liver injury in the United States (Larson et al., 2005). APAP, also known as N-acetyl-p-aminophenol, or paracetamol, is one of the most widely used over-the-counter analgesics and antipyretics and is considered safe at recommended therapeutic concentrations. However, excessive APAP can cause severe liver dysfunction, which threatens patients’ lives (Buckley et al., 1999). Many studies have shown that Nrf2 KO mice are highly sensitive to APAP treatment compared to controls, and the cause of death is the inability to supply glutathione, a product downstream of Nrf2, promptly (Chan et al., 2001; Enomoto et al., 2001). Meanwhile, it has been reported that some protective agents could reduce mitochondrial oxidative stress, the inflammatory response by inhibiting signaling axes such as Keap1/Nrf2/HO-1, and thus protecting hepatocytes from APAP-induced injury (Wang et al., 2019a; Lv et al., 2019; Wu et al., 2019). Among them, corilagin and farrerol, the common hepatoprotective preconditioners, can effectively protect liver cells from APAP-induced acute liver failure (ALF) by activating Nrf2 or upregulating the AMPK/GSK3β-Nrf2 signaling pathway, which in turn is involved in the prevention of hepatotoxicity (Wang et al., 2019a; Lv et al., 2019).

Meanwhile, the role of NF-κB in hepatoprotection cannot be ignored. Maltol, a food-flavoring agent, was found to mitigate APAP-induced inflammatory responses by restraining the phosphorylation of IκKα, IκKβ, and I-κBα in a dose-dependent manner, which exerted a significant liver protection effect (Wang et al., 2019b). And one study demonstrated that corilagin exerted protective effects against APAP-induced hepatotoxicity by inhibiting ERK/JNK mitogen-activated protein kinases (MAPK) and NF-κB signaling pathways (Liu et al., 2020). At the same time, a large number of hepatoprotective reagents (e.g., rutaecarpine, tovophyllin A) have been demonstrated to have significant therapeutic effects by up-regulating Nrf2 transcript levels and inhibiting NF-κB expression (Ibrahim et al., 2018; Choi et al., 2021). In addition, in APAP-induced hepatotoxicity models, the protein levels of Sirt1 are downregulated by IL1β/NF-κB signaling, resulting in inflammation and oxidative stress (Rada et al., 2018). However, the limonin can alleviate APAP-induced hepatotoxicity by activating Nrf2 antioxidative signals and inhibiting NF-κB inflammatory response *via* upregulating Sirt1 (Yang et al., 2020).

#### 4.1.2 Carbamazepine and Azathioprine-Induced Liver Injury

Carbamazepine is a widely used antiepileptic drug that causes acute cholestatic injury and leads to hepatotoxicity. Using the live-cell imaging of the green fluorescent protein (GFP)-based reporter models for Nrf2 and NF-κB signaling, the researchers found that treatment with carbamazepine could cause DILI through an interconnected network of temporal changes in Nrf2 and NF-kB signaling, which influence each other and bring about phenotypes (slightly higher cell death) (Herpers et al., 2016). A study on the gene regulatory networks (GRNs) tells us that in azathioprine-induced liver injury, Nrf2 is widely activated and the activity of NF-κB is inhibited. At the same time, the inferred network reveals the overlap of regulatory genes between NF-κB and Nrf2, demonstrating the relationship between NF-κB, Nrf2, and hepatotoxicity (Souza et al., 2017).

#### 4.1.3 Isoniazid (INH)-Induced Liver Injury

Anti-tuberculosis drugs (e.g., INH)-induced hepatotoxicity (ATDH) is a serious problem in tuberculosis treatment and hepatotoxicity management. INH reduced the phosphorylation of ERK1, then prevented Nrf2 translocation into the nucleus, and raised the cytosolic Nrf2 protein levels, thereby inhibiting Hep3B cell cytoprotection produced by the ARE response (Verma et al., 2015). Further studies revealed that INH increased ROS levels, promoted apoptosis, and decreased Nrf2 importer Karyopherin β1 expression levels, thereby inhibiting the Nrf2 pathway (Verma et al., 2015; Verma et al., 2018). Accordingly, some protective candidates may target Nrf2 against INH-triggered hepatotoxicity or ALF. Sagittaria sagittifolia (SSP), a protectant against hepatotoxicity, increased the expression of Nrf2, glutamate-cysteine ligase, and HO-1, whereas that of keap1 was inhibited in INH-caused liver injury models (Wang et al., 2018). Interestingly, a new sight against hepatotoxicity may be to promote the stress activation of Nrf2 (Jia et al., 2019; Zhang et al., 2019). A study observed a significant increase in the mRNA expression of Nrf2 and the promotion of downstream factors such as HO-1 during INH-induced liver injury in zebrafish larvae (Jia et al., 2019). The Nrf2 signaling pathway was activated in the early stage of INH- triggered hepatotoxicity, as a stress compensatory mechanism, but just partially counteracting hepatotoxicity. Then when the liver injury was aggravated, the activation of the Nrf2 pathway was not enough to resist oxidative stress. Instead, a continuous accumulation of Nrf2 in the nucleus leads to more severe oxidative stress (Zhang et al., 2019).

Meanwhile, some results suggested that the expression of inflammatory mediators NF-κB was also upregulated in INH-induced liver injury. In rat models, *pyrrolidine* dithiocarbamate significantly reduced hepatic biochemical and histological damage by inhibiting the activation of NF-κB and oxidative stress caused by INH in a rat model demonstrating its hepatoprotective effect (He et al., 2020). Other hepatoprotective agents have also been shown to exert the same therapeutic effect (Joseph Martin and Evan Prince, 2017). What’s more, other drugs like gallic acid and quercetin attenuate INH-induced liver injury by improving hepatic redox homeostasis through influence on Nrf2 and NF-κB signaling cascades in Wistar rats (Sanjay et al., 2021a; Sanjay et al., 2021b). So in conclusion, many drugs protect against anti-tuberculosis-induced liver injury by activating Nrf2 and blocking NF-κB, and it is of greater clinical significance to investigate the interaction between Nrf2 and NF-κB.

#### 4.1.4 Triptolide (TP)-Induced Liver Injury

TP is one of the main biological activities and active ingredients of Tripterygium *wilfordii Hook F*, which has excellent immunoregulatory and anti-tumor activities and can be used to treat nephrotic syndrome, cancer, rheumatoid arthritis, and other autoimmune inflammatory diseases (Li et al., 2014a; Li et al., 2015). However, the widespread use of TP remains limited by the narrow therapeutic window and hepatotoxicity (Li et al., 2014a; Xi et al., 2017). To better understand the causes of TP-induced hepatotoxicity, we focused specifically on the role of Nrf2 and NF-κB in TP-induced hepatotoxicity, which also provides direction for future therapeutic targets. MiR155, a regulator of the Nrf2 antioxidant pathway, was significantly activated and then inhibited the activity of the Nrf2 pathway in TP-induced hepatotoxicity (Li et al., 2021a). TP indeed decreased the protein expressions of the Nrf2 pathway including HO-1, NQO1, and Nrf2 associated with the oxidative stress pathway (Zhou et al., 2020). And a study found that TP-induced oxidative stress and cell damage in HepG2 cells could be aggravated by Nrf2 knockdown or counteracted by overexpression of Nrf2 (Li et al., 2014b). In addition, treatment with sulforaphane (SFN), a typical Nrf2 agonist, attenuated TP-induced toxic effects such as liver dysfunction, structural damage, and glutathione depletion in mice by activating Nrf2 and its downstream targets (Li et al., 2014b). To illustrate a deeper mechanism, some scholars have found that catalpol (CAT), the main active ingredient of Rehmannia Glutinosa (RG), enhanced Nrf2 transcript expression by reversing TP-induced Nrf2 transcriptional repression. The idea further proved that CAT induced UGT1A6, a phase II detoxifying enzyme of TP, through the Nrf2 pathway, thereby increasing TP metabolic conversion and reducing TP hepatotoxic effects (Fu et al., 2020). Meanwhile, another agent panax notoginseng saponins (PNS) significantly increased Nrf2 phosphorylation for relieving the potential inhibition of TP-induced Nrf2/ARE binding activity. These results highlight the necessity of the Nrf2/ARE pathway in the TP-triggered hepatotoxicity (Feng et al., 2019).

As an upstream regulator, Nrf2 not only regulates the oxidative stress response but also suppresses inflammation by regulating cytokine production and cross-talk with the NF-κB signaling pathway (Keleku-Lukwete et al., 2018). It has been previously reported that the alleviation of TP-induced hepatotoxicity was involved in the inactivation of the NF-κB pathway (Wei et al., 2017). Besides, TP treatment might disrupt immune homeostasis and induce liver hypersensitivity through the regulation of NF-κB-dependent pro-survival genes (Yuan et al., 2020). These shreds of evidence support the close correlation among unbalanced expression of Nrf2, NK-κB, and hepatotoxicity.

#### 4.1.5 Polygonum *Multiflorum* (PM)-Induced Liver Injury

PM, the root of *P. multiflorum Thunb*, has been used to treat various diseases, such as constipation, early graying of the hair, and hyperlipemia for several decades (Dong et al., 2014). The hepatotoxicity caused by PM treatment has attracted attention in recent years, which was initially considered to be non-toxic (Yu et al., 2011). As one of the main active components of PM, rhein could up-regulate the mRNA and protein levels of Nrf2, HO-1, and glutamate-cysteine ligase catalytic subunit (GCLC), down-regulate the protein expression of NF-κB, TNF-α and caspase-3, increase the survival rate of LO2 cells, decreasing the apoptosis rate and acting as a hepatoprotective drug (Li et al., 2021b). However, there is evidence to confirm that rhein has potential hepatorenal toxicity. A study clarified that immunological idiosyncratic hepatotoxicity induced by PM was associated with inhibition of the NF-κB pathway (Meng et al., 2017). We know that abnormal activation of the TLR4/NF-κB signaling pathway in response to external stimulation can trigger severe liver injury. Also, a large number of results support that PM regulates the Nrf2 pathway, but the specific toxicological mechanism remains to be explored in PM-induced liver injury models (Lin et al., 2018; Wang et al., 2020; Yu et al., 2020).

Overall, Nrf2 and NF-κB interact with each other to coordinate antioxidant and inflammatory responses. There is evidence indicating that Nrf2/HO-1 pathway and NF-κB pathway are involved in olaquindox (OLA)-induced hepatotoxicity. Meanwhile, curcumin can efficiently alleviate OLA-induced hepatotoxicity by further activating Nrf2/HO-1 pathway and inhibiting the p53 and NF-κB pathways to inhibit oxidative stress, apoptosis, and mitochondrial dysfunction (Li et al., 2020a). In the previous study, teicoplanin (TIL) treatment significantly upregulated the relative hepatic expression of HSP70 and NF-κB mRNAs and blocked Nrf2/HO-1 mediated responses, indicating that TIL could induce oxidative stress and hepatotoxicity by blocking Nrf2 mediated defense responses and increasing NF-κB signaling pathways. On the other hand, the combination of astragalus polysaccharide (APS) and TIL improved the expression of the Nrf2 pathway, suggesting that the crosstalk between Nrf2 and NF-κB could be a new therapeutic target against hepatotoxicity (Farag et al., 2019). These pieces of evidence strongly complement our above discussion on the crosstalk of Nrf2 and NF-κB in drug-induced hepatotoxicity, demonstrating that this connection may provide an insight into the development of future hepatoprotective agents.

## 5 Nephrotoxicity

Among the adverse effects, there is a great association between nephrotoxicity and the crosstalk between Nrf2 and NF-κB. Next, we will elaborate in-depth on typical drug-induced nephrotoxicity.

### 5.1 Cisplatin-Induced Nephrotoxicity

Cisplatin, a potent chemotherapeutic agent used for treating various types of solid organ tumors, is limited by its serious side effects in clinical application (Yu et al., 2018; Deng et al., 2020). Cisplatin can cause an increase in the expression of NF-κB and TNF-α in the liver and kidney (Park et al., 2018). And abundant evidence suggests that antioxidant agents promote the anti-inflammatory effect by inhibiting the production of NF-κB in the kidney even in the liver (Taghizadeh et al., 2020; Un et al., 2020). Celastrol, a potent chemotherapeutic agent, could ameliorate cisplatin-induced nephrotoxicity through suppressing NF-κB and protecting mitochondrial function *in vitro* and *in vivo* (Yu et al., 2018). Similarly, polysulfide and H2S could afford protection against cisplatin-caused nephrotoxicity *via* persulfidating STAT3 and IκKβ and inhibiting NF-κB-mediated inflammatory cascade (Sun et al., 2020). In addition, Nrf2 is a key molecule involved in targeting specific proteins in the NF-κB pathway and may be implicated in inflammatory regulation (Deng et al., 2020). Cisplatin can down-regulate Nrf2 and HO-1 while upregulating inflammatory factors. And embelin, an anti-inflammatory drug, can alleviate cisplatin-induced nephrotoxicity by activating the signaling pathway of Nrf2/HO-1 and impeding NF-κB (Qin et al., 2019). Some studies have found that vinorelbine protects against cisplatin-induced acute kidney injury (AKI) by activating Nrf2/HO-1 signaling pathway and hindering TLR4-IFN-γ-CD44 cell inflammatory cascade when investigating the molecular mechanism of renal protection against nephrotoxicity (El-Sayed et al., 2021). Accumulating evidence suggests that the antioxidant mechanism protecting against enucleation-induced nephrotoxicity involves the Nrf2/HO-1 signaling pathway (Younis et al., 2020).

Some scholars have found that in the cisplatin-induced AKI mice, the C. cicadae mycelium extract regulated the inflammatory response by inhibiting renal pathological changes, inflammatory cell infiltration, and the release of a variety of inflammatory cytokines such as TNF-α, IL-1β, and IL-6. Further data revealed that the C. cicadae mycelium extract exerted potent detoxifying effects by inhibiting TLR4/NF-κB/MAPK and regulating the HO-1/Nrf2 signaling pathway while regulating autophagy and inhibiting apoptosis to alleviate renal injury (Deng et al., 2020). Furthermore, thymoquinone and curcumin combination also prevented cisplatin-induced kidney injury by attenuating NF-κB, KIM-1 and ameliorating Nrf2/HO-1 signaling (Al Fayi et al., 2020).

### 5.2 Aristolochic Acid (AA)-Induced Nephrotoxicity

Ingestion of Chinese herbal medicines containing AA can induce aristolochic acid nephropathy (AAN) which is a rapidly progressive tubulointerstitial disease (Vanherweghem et al., 1993). Earlier evidence supports that AA-induced AKI is associated with impaired activation of Nrf2 and its downstream target gene expression (Wu et al., 2014). Therefore, we boldly guess that nephrotoxicity can be alleviated by activating the Nrf2 signaling pathway and increasing the expression of downstream target genes. Unsurprisingly, enhancement of the Nrf2 signaling pathway ameliorated AA-induced tubular epithelial cell injury, while down-regulation of the Nrf2 signaling pathway caused the opposite results, fully demonstrating that Nrf2 plays an important role in the AA-induced nephrotoxicity (Huang et al., 2020). Furthermore, a study has found that methylbarbadenolone (BARD), an antioxidant modulator, could play a renoprotective role through the Nrf2 signaling pathway (Song et al., 2019). It significantly ameliorated AA-induced tubular necrosis and interstitial fibrosis by up-regulating the renal expression of Nrf2 and Smad7 (Song et al., 2019).

In addition, NF-κB is also significantly activated in AA-induced nephropathy (Zhao et al., 2015). Notably, AA exhibited cytotoxicity in a dose-dependent and time-dependent manner. At a safe dose, AA could significantly inhibit the activity of NF-κB in HK2 cells and show the pharmacological effect (Chen et al., 2010). However, in the AA-induced renal injury model, the expression of NF-κB was abnormally elevated, suggesting that the dual effects of NF-κB may be one reason for the different properties of AA on the kidney. Demonstrating our idea from the reverse aspect, the Chinese herb Sedum sarmentosum extract (SSB) reduced the activity of the NF-κB signaling pathway when exerting renoprotective effects (Bai et al., 2014). Also, AA may be involved in the alteration of matrix homeostasis during renal fibrosis *in vivo*, including the imbalance of extracellular matrix (ECM) accumulation and matrix metalloproteinase (MMP) activation involving NF-κB (Tsai et al., 2014). Therefore, we can improve renal fibrosis by regulating the activity of the NF-κB pathway, and providing new ideas for drug therapy of nephropathy (Tsai et al., 2014). All the above evidence suggests that in AA-induced nephrotoxicity, NF-κB is a network core molecule that can modulate the immune and inflammatory responses that occur in the kidney.

### 5.3 Contrast-Induced (CI) AKI

As the third most common cause of AKI, the use of contrast agents and contrast-induced nephropathy (CIN) have become the focus of global public health attention (Yang et al., 2015). This event has been reported to occur in about 30% of patients receiving invasive image examinations and is strongly associated with a high risk of death, long hospital stays, and adverse outcomes such as a high risk of chronic kidney disease (Nash et al., 2002; Finn, 2006). In exploring possible therapeutic options for CIN, researchers have found that a renal protective drug could increase the activation of the Nrf2 antioxidant defense pathway (Khaleel et al., 2017). Further mechanism elaboration, the protective effect against CIN could be exerted through Nrf2/Sirt3/SOD2 or Nrf2/HO-1 pathways (Khaleel et al., 2019; Zhou Q et al., 2019). Moreover, recent studies have found that STC1, a conserved glycoprotein with anti-apoptosis and function, could target Nrf2 to reduce kidney injury and provide a promising preventive target for the treatment of CI-AKI (Zhao et al., 2020).

Similarly, NF-κB is aberrantly activated in CIN, but NF-κB expression is decreased and renal tissue hyalinization, hemorrhagic casts, and necrosis are reduced after infliximab treatment (Saritemur et al., 2015). And TLR4/Myd88/NF-κB is also involved in the protective pathway (Yue et al., 2017). In addition, a study has revealed that overexpression of miR429 inhibits the NF-κB signaling pathway by targeting programmed cell death 4 (PDCD4) to reduce apoptosis and improve cell viability in a CI-AKI cell model (Niu et al., 2021).

### 5.4 Gentamicin (GM)-Induced AKI

GM is an aminoglycoside antibiotic commonly used to treat acute gram-negative bacterial diseases. However, at least 30% of patients who received GM for more than 7 days had an increased risk of AKI (Cuzzocrea et al., 2002). Melatonin (MT) restores antioxidant enzyme activity and blocks NF-κB and nitric oxide synthase (iNOS) activation in rat kidneys, thereby preventing GM-induced nephrotoxicity, and these experimental results confirm that MT protects the kidney as a potent antioxidant (Lee et al., 2012). Besides, GM was found to promote nuclear NF-κB (p65) expression and inflammatory cytokine cytokines (TNF-α and IL-6) and upregulate NF-κB-DNA-binding activity in kidney cells. In contrast, nephroprotective drugs can attenuate the abnormity of these genes, thereby reducing the degree of histological damage and neutrophil cell infiltration in renal tubules (Ansari et al., 2016). Also, NF-κB inhibitors have the same effect (Tugcu et al., 2006). On the other side, both Keap1/Nrf2/ARE and PKC/Nrf2 antioxidant pathways can be activated to enhance the attenuation function of the kidney (Arjinajarn et al., 2016; Ali et al., 2021). In addition, Nrf2 and NF-κB pathways can be simultaneously regulated in GM-induced nephrotoxicity models, and which provides direct evidence between the crosstalk of Nrf2 and NF-κB and drug-induced toxicity (Kalayarasan et al., 2009; Mahmoud, 2017). In future therapeutic strategies, increasing the nuclear immunoreactivity of Nrf2 and decreasing that of NF-κB could be taken into account.

### 5.5 Methotrexate (MTX)-Induced AKI

MTX is an anti-proliferative folic acid antagonist widely used in clinical treatment for cancers and chronic inflammatory diseases, but its nephrotoxicity limits its use. Some researchers have found that MTX-induced nephrotoxicity could be attenuated by vincamine, playing a protective role in the already peripheral and central nervous systems of the kidney. The therapeutic effect of vincamine against MTX-induced renal injury was through inhibiting oxidative stress while decreasing the expression of the NF-κB pathway by increasing the expression of Nrf2 and HO-1 (Shalaby et al., 2019). And some other nephroprotective agents, such as chicoric acid, umbelliferone and berberine, also exert their protective effects by the same mechanism (Hassanein et al., 2018; Abd El-Twab et al., 2019; Hassanein et al., 2019). Indeed, the importance regarding the modulation of NF-κB and Nrf2 pathway activity in MTX-induced AKI has been efficiently shown (Sherif et al., 2020), especially Nrf2. Activation of the Nrf2/HO-1 signaling pathway can effectively protect kidney structure from destruction, while various factors lead to the elevated activity of Nrf2/HO-1, such as the expression level of miR145-5p which directly targets Sirt5 (Mahmoud et al., 2018; Aladaileh et al., 2019; Li et al., 2021c).

Let us pay attention to whether, the body affected by nephropathy also causes further nephrotoxicity by breaking the balanced relationship between the two pathways, in addition to nephrotoxicity caused by the crosstalk of Nrf2 and NF-κB. When exploring whether diabetes aggravates I/R-induced AKI in rats, some results have shown that diabetes aggravated oxidative stress, inflammatory response, and apoptosis after renal I/R by enhancing TLR4/NF-κB signaling and blocking the Nrf2/HO-1 pathway. However, pretreatment with tBHQ (an Nrf2 agonist) inhibited NF-κB signaling and enhanced the function of anti-apoptosis (Gong et al., 2019). These results indicate that the crosstalk between Nrf2 and NF-κB is involved in the development of nephrotoxicity.

## 6 Cardiotoxicity

As same as nephrotoxicity, there are lots of results that have shown a tight association between the crosstalk and drug-induced cardiotoxicity.

### 6.1 Doxorubicin (DOX)-Induced Cardiotoxicity

DOX, an anthracycline antibiotic, has been widely used to treat both solid and hematologic malignancies (Octavia et al., 2012). However, DOX can lead to myocardial cell loss, mitochondrial dysfunction, myelofibrosis, and congestive heart failure in the body, triggering cardiotoxicity after long-term use (Eschenhagen et al., 2011; Brown et al., 2015; Varga et al., 2015; Chung and Youn, 2016; Zagar et al., 2016). DOX-induced cardiotoxicity is mediated by ROS production and the Nrf2/ARE pathway. Natural compounds (NCs), such as Asiatic acid, α-linolenic acid, apigenin, and β-LAPachone, were confirmed to ameliorate DOX-induced cardiac injury by activating Nrf2 in different pathways (Yarmohammadi et al., 2021). For example, cardamonin (CAR), a flavone found in Alpinia plants, can reduce the NF-κB signaling pathway and improve Nrf2 signaling to suppress oxidative stress, apoptosis, and inflammatory response, ameliorating DOX-induced cardiotoxicity (Qi et al., 2020). And previous studies have shown that miR140-5p increases DOX-induced myocardial oxidative damage by inhibiting Nrf2 and Sirt2 signaling pathways (Zhao et al., 2018). Conversely, miR200a protects against DOX-induced cardiotoxicity by activating the Nrf2 signaling pathway (Hu X et al., 2019). This suggests that microRNA acts as a regulatory medium to modulate the Nrf2 pathway to aggravate or ameliorate DOX-induced cardiotoxicity.

Notably, DOX induced the expression of NF-κB p65 and caspase-3 in myocardial nuclei in a DOX-induced cardiotoxicity model. Interestingly, NF-κB p65 and caspase-3 were significantly inhibited by trifluoperazine, a strong calmodulin antagonist, suggesting that the cardioprotective effect conferred by trifluoperazine involves inhibition of NF-κB and apoptosis. Furthermore, biochemical and histopathological examinations revealed that trifluoperazine ameliorated DOX-induced renal and hepatic injury both functionally and structurally (Goda et al., 2021). Similarly, another drug geraniol may function as a potential activator of Nrf2, subsequently improve Nrf2 dependent antioxidant signaling, reduce apoptosis, and attenuate inflammatory responses (Younis et al., 2021). Antioxidant drugs may be a potential therapeutic target to prevent the development of DOX-induced cardiotoxicity by inhibiting oxidative stress and NF-κB pathways (Sahu et al., 2019; Xu et al., 2020).

The study suggests the necessity and importance of discussing the crosstalk between Nrf2 and NF-κB in drug-induced cardiotoxicity. On top of that, among the organ injuries, the rising morbidity and mortality of highlighting cardiovascular diseases (CVDs) make cardiac injury attract people’s attention. Some studies have found that aging is a major risk factor for CVDs (Niccoli and Partridge, 2012; Jaul and Barron, 2017; Franceschi et al., 2018). Under physiological conditions, Nrf2 and NF-κB are degraded *via* the proteasome without cardiotoxicity. When aging disrupts the balance of Nrf2 and NF-κB, it can increase ROS/RNS and activate inflammatory signaling pathways, thereby damaging cellular components and leading to cardiac injury (de Almeida et al., 2020). Also, the ability of adhesion, proliferation, migration, and formation of capillary-like structures is compromised by dysfunction of Nrf2 signaling and it revealed Nrf2 might be a prominent target to release angiogenesis and microvascular rarefaction (Valcarcel-Ares et al., 2012). It has earlier been reported that the NF-κB p65 subunit inhibits the Nrf2/ARE pathway at the transcriptional level (Liu et al., 2008), so there may be crosstalk between NF-κB and Nrf2 in the decrease of Nrf2 expression.

## 7 Pulmonary Toxicity

### 7.1 Bleomycin (BLM)-Induced Lung Injury

BLM, a glycopeptide antibiotic, is an effective antineoplastic agent commonly used to treat breast, ovarian, lung, and different types of leukemia (Levine et al., 1985). However, the effective use of BLM in chemotherapy remains limited, as it precipitates dose-dependent interstitial pneumonia, resulting in pulmonary fibrosis in at least 10% of individuals treated with BLM, ultimately leading to irreversible lung structural damage (Yen et al., 2005; Di Paola et al., 2016). BLM aggravates pulmonary inflammation and apoptosis by up-regulating the expression of NF-κB signaling pathway and apoptosis regulator caspase-3, and substantially reducing the activity of antioxidant enzymes in a rat model of BLM-induced pulmonary fibrosis (Beigh et al., 2017). Furthermore, NF-κB p105 and NF-κB p65 are involved in the progression of acute lung injury (ALI) and play important roles in tumorigenesis, inflammation, and immunity, which are important for elucidating therapeutic strategies for ALI (Shaikh and Prabhakar Bhandary, 2020). Juglans regia extract can inhibit NF-kB activation and reduce the expression of proinflammatory biomarkers such as cyclooxygenase 2 (COX-2) and iNOS to slow down pulmonary fibrosis. The protective effect is to reduce BLM-induced oxidative stress and pulmonary inflammation by regulating the inflammatory response of rat alveolar macrophages (Beigh et al., 2017). Meanwhile, the involvement of the Nrf2 pathway in the process of BLM-induced lung injury has also been mentioned (Liu et al., 2017b). It has become clear that artemisinin, a novel Nrf2 activator, activates the Nrf2 pathway by reducing Nrf2 ubiquitination and improving its stability (Chen et al., 2016). On the other hand, the subsequent antioxidant protection can be achieved by regulating the Nrf2/HO-1 signaling pathway (Ahmad et al., 2020).

The hepatoprotective effects of NF-κB and Nrf2 have been demonstrated in BLM-treated animals. It is curious whether there are potential interaction mechanisms between the two genes that together regulate antioxidant levels to protect against lung injury? Earlier studies have shown that NF-κB and Nrf2 expression levels are simultaneously regulated by hepatoprotective drugs. In BLM-treated pneumonocytes, salidroside inhibits IκBα phosphorylation and the nuclear accumulation of NF-κB p65 while activating Nrf2-antioxidant signaling (Tang et al., 2016). In addition, hesperidin attenuates BLM-induced lung toxicity by inhibiting the IκBα/NF-κB pathway, which in turn improves the regulation of oxidative inflammatory markers (Nrf2 and HO-1) and pro-inflammatory markers (TNF-α, IL-1β, and IL-6) to reduce collagen deposition during pulmonary fibrosis (Zhou Z et al., 2019). Besides, treatment of Glycyl-L-histidyl-L-lysine (GHK)-Cu complexes modulates the Nrf2 and NF-κB pathways, and Smad2/3 phosphorylation partially prevents the development of epithelial-mesenchymal transition (EMT) and has a protective effect against BLM-induced inflammation and oxidative stress. However, the specific interaction mechanism between the two remains to be elucidated (Ma et al., 2020). A study revealed that the activation of NF-κB and Nrf2 pathways played opposite roles in lung injury, respectively. And in the hepatoprotective mechanism, the activity of Nrf2 increases, which has a potential inhibitory effect on the NF-κB pathway, plays a better protective effect (Kang et al., 2020).

## 8 Dermal Toxicity

Psoriasis is a skin disease mediated by immune response disorders, with a prevalence of 2–3% in the world’s population (Brück et al., 2018; Lee et al., 2020). And the patients are suffered from skin itching, pain, seriously affecting the quality of life of patients (Nestle et al., 2009; Boehncke and Schön, 2015). While the etiology of psoriasis remains to be fully elucidated, studies are revealing the crosstalk between Nrf2 and NF-κB in psoriasis (Sangaraju et al., 2021).

### 8.1 Imiquimod (IMQ)- Induced Psoriasis

The results from IMQ-induced psoriasis models *in vitro* confirm the involvement of NF-κB1 in psoriasis pathogenesis through mediated T cell (Th1 and Th17) activation (Zhou et al., 2018). The role of Nrf2 and NF-κB pathways in psoriasis has been previously documented, but the exact interaction between the two has not been elucidated (Li et al., 2020b; Sangaraju et al., 2021). Tussilagonone (TGN), a sesquiterpenoid isolated from Tussilagofarfara, has been used to treat IMQ-induced psoriasis-like dermatitis mice when further exploring transcription factors associated with psoriasis pathogenesis. As a potent Nrf2 activator, TGN can significantly activate the Nrf2 pathway and induce the expression of its downstream target gene HO-1 by inhibiting NF-κB and STAT3. In turn, activation of Nrf2/HO-1 by TGN also inhibits activation of NF-κB and STAT3 which are the key transcription factors in psoriasis pathogenesis and promote the skin defense system while reducing the expression of immune mediators and epidermal proliferation (Lee et al., 2020). Further proving our subject, 2,3,7,8-tetrachlorodibenzo-p-dioxin (TCDD) aggravated IMQ-induced psoriasis by increasing the expression of phosphorylated NF-κB p65 and inhibiting the antioxidant marker Nrf2 (Kim et al., 2021). These studies have provided a novel and clinically meaningful perspective on the pathogenesis and therapeutic targets of psoriasis in the world.

Experimental results from other perspectives have also demonstrated the importance of the Nrf2 and NF-κB pathways in skin inflammation, especially psoriasis. Fumarate esters (FAEs) such as dimethyl fumarate (DMF) and its active metabolite monomethyl fumarate (MMF) can play a vital role in the treatment of psoriasis because of their immunomodulatory effects (Brück et al., 2018). On the one hand, the binding of DMF and MMF to the cysteine residues of Keap1 induces a conformational change that dissociates Nrf2 from Keap1 and enters the nucleus, thereby activating cytoprotective and anti-inflammatory genes (Linker et al., 2011). On the other hand, DMF can also directly or indirectly inhibit the nuclear activity of NF-κB, affecting cytokine production in human T cells, human breast cancer cell lines, and mouse dendritic cells (Gerdes et al., 2007; Ghoreschi et al., 2011; Kastrati et al., 2016).

Interestingly, some researchers have found that synchronized circadian clocks could not only enhance the protective role of Nrf2 but also attenuate the validated response through the NF-κB signaling pathway when testing the differential responses of keratinocytes clock synchronized or desynchronized in challenged keratinocytes by O3. The synchronized circadian clocks can strengthen the cellular defense function and alleviate skin inflammation, which provides new insight into the crosstalk of Nrf2 and NF-κB (Frigato et al., 2020).

## 9 Neurotoxicity

Chemotherapy agents such as oxaliplatin, cisplatin, paclitaxel, and bortezomib frequently cause severe peripheral neuropathy and there is currently no effective strategy to prevent drug-induced toxicity.

### 9.1 Cisplatin-Induced Neurotoxicity

Cisplatin can cause not only nephrotoxicity but also cause severe neurotoxicity, which significantly limits its clinical use. A study showed that a continuous high dose of cisplatin stimulation disrupted the blood-brain barrier, leading to brain cell edema and neuronal necrosis (Namikawa et al., 2000). Regarding the study of prevention and treatment of cisplatin-induced neurotoxicity, the neuroprotective effects exerted by many agents are closely related to the Nrf2 and NF-κB pathways. Schisandrin B (Sch B) has been reported earlier to effectively inhibit the activation of NF-κB, p53 and cleave caspase-3 expression in cisplatin-treated mice, thereby exerting the efficacy of protective drugs (Giridharan et al., 2012). Recently, a large number of articles have found that agents including edaravone and epigallocatechin-3-gallate (EGCG) prevented the development of cisplatin-related brain inflammation and oxidative damage by up-regulating the gene expression level of the Nrf2/HO-1 pathway and preventing the cisplatin-induced NF-κB activation (Jangra et al., 2016; Arafa and Atteia, 2020).

### 9.2 Bortezomib-Induced Neurotoxicity

Bortezomib was the first proteasome inhibitor approved to treat multiple myeloma (MM), mantle cell lymphoma, and other solid tumors. However, peripheral neuropathy (PNP) is one of the most common side effects of bortezomib and leads to dose modification and discontinuation of therapy. One hallmark of the mechanism of action of bortezomib in tumor cells is NF-κB, while bortezomib treatment may induce neuropathy by inhibiting NF-κB in non-neuronal types or targeting other signaling pathways. It has been found that animals with impaired NF-κB activation developed significantly less neuropathy, suggesting that the NF-κB activation played an active role in bortezomib-induced neuropathy (Alé et al., 2016). There was also evidence to identify the NF-κB signaling pathway mediating neurotoxicity when investigating the molecular factors associated with PNP in patients with MM induced by bortezomib (Campo et al., 2017). Thus, inhibition of NF-κB may be a promising strategy to protect against bortezomib-triggered neurotoxicity.

### 9.3 Paclitaxel (PTX) and Qxalipatin-Induced Neurotoxicity

PTX is an anti-tumor drug with high efficacy, especially for breast cancer, ovarian cancer, bladder cancer, lung cancer, and other types of solid tumors (Melli et al., 2006; Malekinejad et al., 2016). Despite its dramatic effect, PTX can cause severe peripheral neuropathy (usually manifested as painful neuropathy), affecting up to 50% of cancer patients (Hu L. Y et al., 2019). Huangqi Guizhi Wuwu Decoction (HGWD) was found to have a therapeutic effect on oxaliplatin-induced peripheral neuropathy. It was revealed that HGWD significantly inhibited PTX -induced activation of TLR4/NF-κB and decreased the expression of IκKα, and phosphorylation of NF-κB to inhibit PTX-evoked inflammatory and oxidative responses in the peripheral nervous system. It also promoted activation of the PI3K/Akt-Nrf2 signaling pathway, alleviating PTX-induced oxidative stress to some extent (Lv et al., 2021). Moreover, other protective drugs have also been shown to regulate the downstream Nrf2/HO-1 pathway (Yardım et al., 2021). Another chemotherapeutic drug, oxaliplatin, also often causes peripheral neuropathy afflicting patients. However, some studies have found that L-carnosine exerted a neuroprotective effect against oxaliplatin-induced peripheral neuropathy in colorectal cancer patients *via* the Nrf2 and NF-κB pathways (Yehia et al., 2019).

According to the results of another study, we reasonably hypothesize that the protective mechanism against chemotherapy-induced neurotoxicity may be that protective drugs and their metabolites increase the DNA-binding activity of Nrf2, thereby enhancing the ability to counteract oxidative stress (Kawashiri et al., 2018). On the other hand, the potential inhibitory effect of Nrf2 on NF-κB inhibits the inflammatory response and enhances the alleviation of neurotoxicity. However, it is noteworthy that elevation of Nrf2 signaling by protective agents can also activate the phase I, II and III response in tumors. Increased expression of xenobiotic metabolism and efflux pumps due to compounds (protective agents mentioned above) that elevate Nrf2 in cancer maybe diminish anti-cancer agent efficacy. These concerns may be diminished due to co-administration agents possessing, such as pharmacokinetic properties which cause differential temporal effects, and treatment regimens that cause increased metabolism/efflux transporter expression only after anti-cancer agents have already bound to their target. Other effects such as organ-specific compound uptake and other variables could also be explained. Notably, differences between genetic and pharmacologic activation of Nrf2 signaling also exist (Kensler and Wakabayashi, 2010).

### 9.4 The Crosstalk Between Nrf2 and NF-κB in Neurodegenerative Disorders

Suppression of Nrf2 can cause the development of neurotoxicity in animal models (Sandberg et al., 2014). Previous studies have reported that Nrf2 deficient mice developed vacuolar (spongiform) leukoencephalopathy along with extensive astrogliosis, revealing a possible physiological role for Nrf2 in maintaining myelin in the central nervous system (Hubbs et al., 2007). A dysfunctional Nrf2 system may increase the risk of chronic diseases in humans, such as Alzheimer’s disease (AD) and Parkinson’s disease (PD). A study in neurodegenerative disease showed that the amount of Nrf2 was decreased in hippocampal astrocytes which were one of the brain regions where neurodegeneration began in AD patients. Also, in PD patients, the nuclear localization of Nrf2 is strongly induced, but this response may not be sufficient to protect neurons from degeneration (Ramsey et al., 2007). In addition, genetic variants in the Nrf2 gene have been associated with the progression of AD and PD (von Otter et al., 2010a; von Otter et al., 2010b). Several studies have shown that NF-κB activation in astrocytes could aggravate neuroinflammation and produce neurotoxic effects (Schwaninger et al., 1999; Acarin et al., 2001; de Freitas et al., 2002; Brambilla et al., 2005). In addition, the expression of various NF-κB target genes and NF-κB DNA-binding activity increased in the brains of AD patients (Hensley, 2010). Several genetic, cell biology, biochemical and animal studies supported that inhibiting NF-κB could play a critical role in preventing the development of AD pathology (Selkoe, 2001; Ghiso and Frangione, 2002).

Consistent with the above content, a study showed that DMF is involved in protecting β-amyloid-induced cytotoxicity and alleviating neurotoxicity by reducing the pro-inflammatory pathway: NF-κB, as well as regulating the Nrf2 pathway to increase the production of antioxidant enzymes (Campolo et al., 2018). A study found that DMF exerted neuroprotective effects through the crosstalk between Nrf2 and NF-κB pathways *in vitro* and *in vivo,* besides regulating antioxidant enzymes such as Nrf2, Mn-SOD, and HO-1, thereby reducing the levels of pro-inflammatory cytokines such as IL-1 and iNOS (Campolo et al., 2017). The study also has elucidated the crosstalk between Nrf2 and NF-κB pathways in acrylamide-induced neurotoxicity and found that the protective effect of oxidant N-Acetyl-L-Cysteine (NAC) against acrylamide was to resist cell injury by activating the Nrf2 pathway and inhibiting NF-κB pathway. In addition, MAPKs, as the central mediators that transmit extracellular signals from cell membrane to nucleus, can regulate the activation of Nrf2 and NF-κB pathways to reduce cell injuries (Pan et al., 2018). Vincristine was proved to increase NF-κB levels in sciatic nerves and consequently cause oxidative stress and neuroinflammation through the suppression of the Nrf2 pathway. Meanwhile, the quercetin treatment increased Nrf2 levels and downregulated NF-κB levels to alleviate oxidative stress and neuroinflammation (Yardim et al., 2020). Another research highlights that acrylamide, a common food contaminant formed during food heat processing, induces oxidative stress in astrocytes and microglia by regulating the Nrf2/ARE and NF-κB pathways, leading to neurotoxicity. These results reveal the importance of the crosstalk of Nrf2 and NF-κB signal pathways in neurotoxicity (Zhao et al., 2017).

Furthermore, it has been proved that mice bearing Nrf2 deficiency were suffered from neuroinflammation and lost dopaminergic neurons (Lastres-Becker et al., 2012). By comparison, the neuroprotection appeared in a mutant alpha-synuclein (αSyn; a small protein with 140 amino acids abundant in presynaptic nerve terminals) transgenic mouse model with an overexpression level of Nrf2 gene (Gan et al., 2012). At the same time, the increased activation of NF-κB is related to the strong nuclear p65 immunoreactivity in PD patients, which suggests that NF-κB participates in neurodegenerative disorder (Mattson and Camandola, 2001). Also, evidence has shown that we could take NF-κB as an ideal therapeutic target due to its critical role in forming inflammatory mediators in models of PD-induced neurotoxicity (Flood et al., 2011). Thus, it is obvious that Nrf2 and NF-κB interplay in neurodegenerative disorders, in which the increase of NF-κB aggravates neuroinflammation while the increase of Nrf2 affords neuroprotection (Sivandzade et al., 2019).

## 10 Therapeutical Strategies for Drug-Induced Adverse Reactions

Here, we summarize the protective agents that may alleviate injury in different organ toxicities as well as their effects in anti-oxidative and anti-inflammatory systems (Table 1).

**TABLE 1:** A summary of representative protective agents targeting the crosstalk between the Nrf2 and NF-κB pathway in drug-induced toxicity.

Toxic reaction	Representative drug	Representative promising candidate	Brief summary of regulatory mechanisms based on Nrf2 and NF-κB	References
Hepatotoxicity	Acetaminophen	Salvianolic acid B/C	• Regulating drug-metabolizing enzymes, transporter and antioxidant genes through the Nrf2/ARE pathway	Wang et al. (2019a), Lv et al. (2019), Wu et al. (2019), Wang et al. (2019b), Ibrahim et al. (2018), Rada et al. (2018), Yang et al. (2020), Choi et al. (2021), Fan et al. (2014), Jiet al. (2015), Fan et al. (2018), Lv et al. (2018), Lv et al. (2020), Zhang et al. (2020)
		Corilagin; Isoorientin	• Mitigating mitochondrial oxidative stress, inflammatory response, and caspase-mediated antiapoptotic effect through inhibition of Keap1/Nrf2/HO-1 axis	
		Farrerol; Tanshinone IIA	• Exerting antioxidant effects through Nrf2 activation *via* the AMPK/AKT pathway	
		Maltol; Wuzhi tablet	• Upregulating AMPK/GSK3β-Nrf2 signaling pathway	
		Rutaecarpine; Quercetin	• Inhibiting oxidative stress and inflammation response *via* NF-κB and PI3K/Akt pathway	
		Tovophyllin A	• Activating the Nrf2 pathway and inhibiting NF-κB inflammatory response *via* upregulating Sirt1	
		Limonin; Daphnetin	• Regulating Nrf2/Trx-1 axis *via* decreasing ASK1/JNK and Txnip/NLRP3 inflammasome	
		Licochalcone A		
	Isoniazid	Gallic acid; Quercetin	• Increasing the expression of antioxidant enzymes and decreasing CYP expression through the activation of the Nrf2/Keap1 pathway	Wang et al. (2018), Jia et al. (2019), Zhang et al. (2019), He et al. (2020), Sanjay et al. (2021a), Sanjay et al. (2021b)
		Pyrrolidine dithiocarbamate	• Increasing BSEP expression, and exhibiting + antioxidant and anti-inflammatory activities	
		Sagittaria sagittifolia	• Improving the redox homeostasis by activating Nrf2 and blocking NF-κB/TLR-4 axis	
	Triptolide	Arctiin; Quercetin; Licorice	• Acting as an upstream activator to regulate Nrf2 and its downstream target (HO1, NQO1)	Zhou et al. (2020), Feng et al. (2019), Fu et al. (2020), Wei et al. (2017), Yuan et al. (2020), Li et al. (2014b), Hou et al. (2018)
		Panax notoginseng saponins	• Inducing phase I/II detoxification enzyme *via* Nrf2 pathway to increasing metabolic conversion	
		Sulforaphane; Catalpol	• Restoring Th17/Treg balance through Tim-3 and TLR4-MyD88-NF-κB pathway	
	Polygonum multiflorum	Pioglitazone	• Decreasing serum TNF-α and other inflammatory cytokines, liver tissue PPAR-γ expression, and inhibiting expression of NF-κB p65	Meng et al. (2017)
Nephrotoxicity	Cisplatin	*Cordyceps cicadae* Mycelia	• Decreasing phosphorylation of STAT3 and IκKβ STAT3 and IκKβ, inhibiting NF-κB-mediated inflammatory cascade and improving mitochondrial function	Yu et al. (2018), Deng et al. (2020), Qin et al. (2019), Al Fayi et al. (2020), Sun et al. (2020), Taghizadeh et al. (2020), Younis et al. (2020), El-Sayed et al. (2021)
		Vincamine; Embelin; Polysulfide	• Diminishing oxidative stress and inflammation by activating the signaling pathway of Nrf2/HO-1 and impeding NF-κB	
		Celastrol; Gliclazide; Vanillin		
		Thymoquinone and Curcumin		
	Aristolochic Acid	Bardoxolone methyl	• Upregulating Nrf2/Smad7, NQO1, and HO-1 expression and downregulating Keap1 expression	Wu et al. (2014), Song et al. (2019), Bai et al. (2014)
		Sedum sarmentosum extract	• Decreasing the activity of the NF-κB signaling pathway, resulting in down-regulated expression of NF-κB-controlled chemokines and pro-inflammatory cytokines	
	Contrast	Atorvastatin; Infliximab; stanniocalcin-1	• Activating the Nrf2/Sirt3/SOD2 or Nrf2/HO-1 pathways	Saritemur et al. (2015), Khaleel et al. (2017), Yue et al. (2017), Khaleel et al. (2019), Zhou Z et al. (2019), Zhao et al. (2020)
		Lansoprazole; Sulforaphane	• Suppressing the TLR4/Myd88 pathway and inhibiting the expression of downstream inflammatory cytokines, such as IL-1β, TNF-α, IL-6, and MCP-1	
		*Tert*-Butylhydroquinone	• Alleviating oxidative DNA damage, mitochondrial damage and apoptosis *via* the Nrf2 pathway	
	Gentamicin	Diallyl sulfide; Diosmin	• Restoring antioxidant enzyme activity, and blocking NF-κB and iNOS activation	Tugcu et al. (2006), Kalayarasan et al. (2009), Lee et al. (2012), Ansari et al. (2016), Arjinajarn et al. (2016), Mahmoud (2017), Ali et al. (2021)
		Melatonin; Kiwi fruit	• Upregulating nuclear NF-κB p65 expression, NF-κB-DNA binding activity, and MPO activity	
		Pyrolidium dithiocarbamate	• Activating Keap1/Nrf2/ARE, AKT or PKC/Nrf2 antioxidant pathways	
		sulfasalazine; Sinapic acid		
		Riceberry bran extract		
	Methotrexate	Berberine; Chicoric acid; Dioscin	• Activating Keap1/Nrf2 signaling and attenuating ROS-induced activation of NF-κB/NLRP3 inflammasome signaling and apoptosis pathways	Hassanein et al. (2018), Mahmoud et al. (2018), Abd El-Twab et al. (2019), Aladaileh et al. (2019), Hassanein et al. (2019), Shalaby et al. (2019), Sherif et al. (2020), Li et al. (2021c)
		Commiphora molmol; Vincamine	• Regulating antioxidant pathways and anti-inflammatory pathways *via* miR145-5p or miR29a	
		Formononetin; Umbelliferone		
Cardiotoxicity	Doxorubicin	Asiatic acid; Apigenin; Geraniol	• Activating the Nrf2/Keap1/ARE pathway	Qi et al. (2020), Yarmohammadi et al. (2021), Sahu et al. (2019), Goda et al. (2021), Younis et al. (2021)
		Cardamonin; Baicalein; Dioscin	• Inhibiting oxidative stress, MAP kinase activation, NF-κB pathway, PI3K/Akt/mTOR impairment, and cardiac apoptosis	
		sulforaphane; tanshinone IIA		
		Trifluoperazine; Wheat phenolics		
Pulmonary Toxicity	Bleomycin	Artemisitene; Curcumin	• Activating Nrf2 by reducing Nrf2 ubiquitination and improving its stability and inhibit of NF-κB and TGF-β1/Smad2/3/AMPK pathways	Chen et al. (2016), Tang et al. (2016), Beigh et al. (2017), Liu et al. (2017b), Zhou Z et al. (2019), Ahmad et al. (2020), Ma et al. (2020), Shaikh and Prabhakar Bhandary (2020)
		Hesperidin; Pirfenidone	• Inhibiting the IκBα/NF-κB pathway, which in turn improves the regulation of oxidative inflammatory markers (Nrf2 and HO-1) and proinflammatory markers (TNF-α, IL-1β, IL-6, COX-2, and iNOS)	
		Glycyl-L-histidyl-l-lysine		
		Salidroside; Thymoquinone		
		Walnut extract		
Dermal toxicity	Imiquimod	Dimethylfumarate	• Attenuating psoriasis-related inflammatory, regulating cellular anti-oxidant responses and suppression of keratinocyte hyperproliferation *via* activation of Nrf2/HO1 and inhibition of NF-κB and STAT3	Lee et al. (2020), Sangaraju et al. (2021), Ogawa et al. (2020)
		Tussilagonone (TGN)	• Influencing cytokine production by antigen-presenting cells, inhibiting Th1/Th12 responses, promoting Th2 responses *via* indirect and/or direct inhibition of NF-κB	
		Galangin		
Neurotoxicity	Cisplatin	Epigallocatechin Gallate	• Inhibiting NF-κB and p53 activation and upregulating Nrf2/HO-1 pathway	Giridharan et al. (2012), Jangra et al. (2016), Arafa and Atteia (2020)
		Edaravone; Schisandrin B		
	Bortezomib	Dimethyl fumarate	• Activating the Nrf2 pathway	Kawashiri et al. (2018)
	Paclitaxel and Qxalipatin	Curcumin; Dimethyl fumarate	• Inhibiting activation of the inflammatory TLR4/NF-κB pathway and promoting the activation of PI3K/Akt-Nrf2 signaling pathway	Kawashiri et al. (2018), Yehia et al. (2019), Lv et al. (2021), Yardım et al. (2021)
		Huangqi Guizhi Wuwu Decoction (HGWD)	• Protecting neural stem/progenitor cells and neurons from oxidative damage through the Nrf2-ERK1/2 MAPK pathway	
		L-Carnosine		

## 11 Perspectives and Conclusion

**FIGURE 4:**
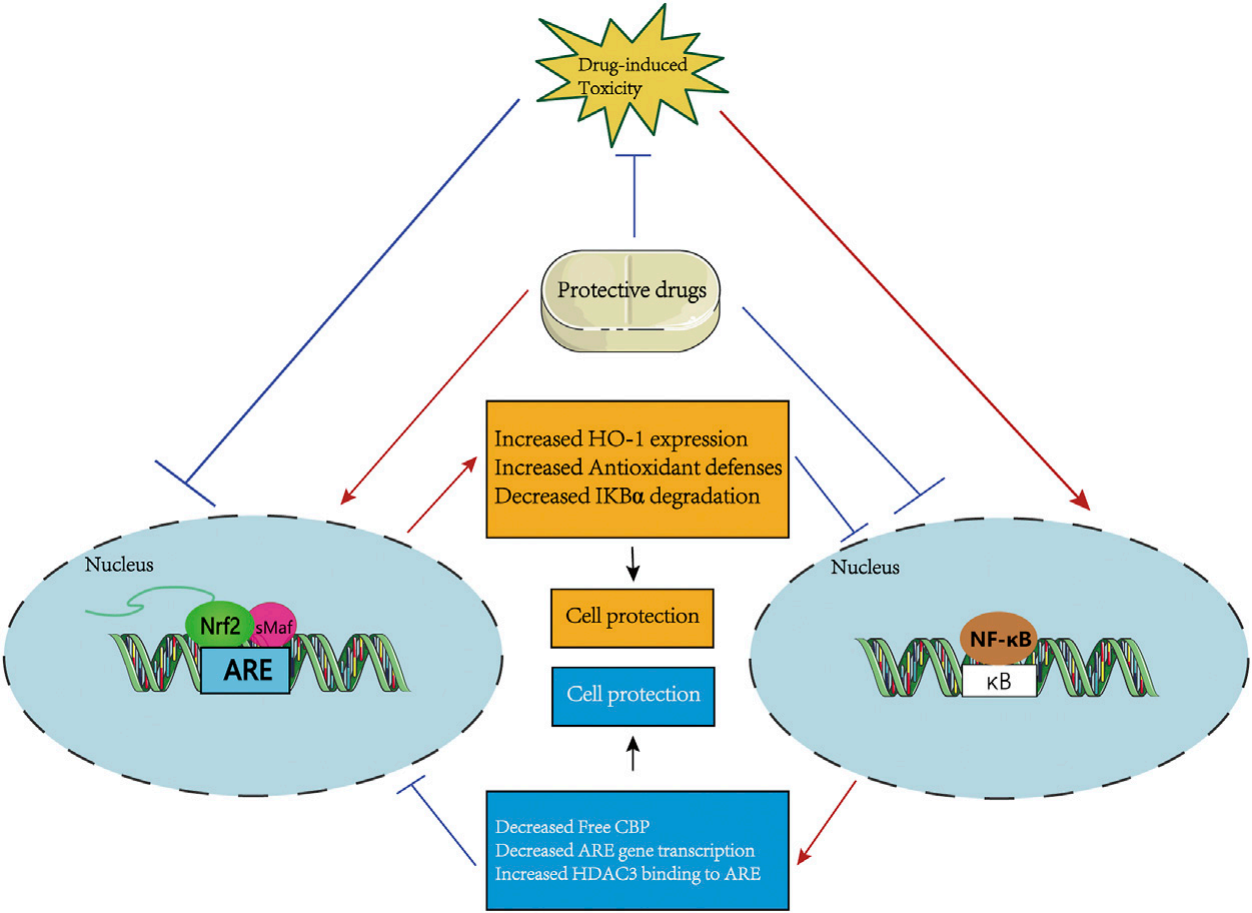
A systematic schematic of protective agents against drug-induced toxicity *via* modulating the mutual interference between Nrf2 and NF-κB pathways. The protective candidates improve the antioxidant capacity by activating the Nrf2 pathway and inhibiting the NF-κB-mediated inflammatory response, thereby antagonizing the drug-induced organ toxicity. Specifically, on the one hand, the protective agents directly or indirectly activate the Nrf2 signaling, then prevent IκBα degradation, or increase HO-1 expression to inhibit NF-κB activation, thereby increasing antioxidant defense ability to resist drug-induced toxicity. On the other hand, the protective agents may inhibit the nuclear translocation of NF-κB which activating by drug-induced toxicity to increase the activation of the Nrf2 pathway by increasing ARE gene transcription and Free CBP, and reducing the recruitment of HDAC3 to the ARE region.

In clinical applications, the treatment of many drugs, including APAP and antineoplastic agents, is limited by their potential organ toxicity. However, the mechanism of drug-induced tissue injury and the corresponding protective strategies have not been fully discussed. Meanwhile, there is increasing interest in investigating the relevance of Nrf2 and NF-κB signaling pathways in the development/progression of drug-induced injury. Therefore, this review summarizes and discusses the role of common agents in the induction of multiple organ toxicities. We found that the unbalanced relationship between Nrf2 and NF-κB may induce or aggravate toxic responses, while substances that regulate the relationship between Nrf2 and NF-κB may play a protective role-restoring Nrf2 activity, thereby enhancing antioxidant capacity and reducing NF-κB-mediated inflammatory responses. The crosstalk of both may serve as a central hub for drug-induced toxic responses. At the same time, drugs or therapeutic approaches that target the crosstalk between Nrf2 and NF-κB appear to be promising pointcuts for future clinical responses to drug-induced toxicity (Figure 4). In addition, further study and therapeutic exploration of potential molecular mechanisms will help explain the specific regulatory mechanisms of Nrf2 and NF-κB signaling pathways in various drug-induced organ toxicities, and ultimately standardize rational and safe medicine usage and reduce the occurrence of toxic reactions. In summary, we urgently need signaling network studies of Nrf2 and NF-κB in pathological situations, as well as new treatments and new drugs targeting the crosstalk between the two.
